# Efficacy and safety of acupuncture in the treatment of chemotherapy-induced peripheral neuropathy in breast cancer patients: a systematic review and meta-analysis

**DOI:** 10.3389/fneur.2025.1690446

**Published:** 2026-01-14

**Authors:** Bozhen Huang, Meijiao Zhou, Shanshan Song, Siyi Ma, Min Jiang

**Affiliations:** 1 Department of Traditional Chinese Medicine, Beijing Shijitan Hospital, Capital Medical University, Beijing, China; 2 Department of Rheumatology and Immunology, First Teaching Hospital of Tianjin University of Traditional Chinese Medicine, Tianjin, China

**Keywords:** acupuncture, breast cancer, chemotherapy, meta-analysis, peripheral neuropathy

## Abstract

**Background:**

Chemotherapy-induced peripheral neuropathy (CIPN) is a common and debilitating side effect in breast cancer survivors. This meta-analysis evaluates the efficacy and safety of acupuncture for CIPN management.

**Methods:**

We systematically searched PubMed, Embase, Web of Science, Cochrane Library, China National Knowledge Infrastructure, Wanfang Database, VIP Database, and Chinese Biomedical Literature Database from database inception to August 3, 2025, for randomized controlled trials (RCTs) on acupuncture treatment for CIPN in breast cancer patients. We used RevMan 5.2 and Stata 16.0 for meta-analysis.

**Results:**

A total of 10 RCTs involving 653 patients were included. Treatment group significantly improved the clinical efficacy versus control group (RD = 0.22, 95% CI: 0.10, 0.33; *p* < 0.001). Chemotherapeutic agent subgroup analysis showed that acupuncture was beneficial for taxane-induced CIPN (RD = 0.26, 95% CI: 0.14, 0.38; *p* < 0.001) and utidelone-induced CIPN (RD = 0.33, 95% CI: 0.10, 0.56; *p* = 0.004), while the effect for CIPN from unspecified agents was not statistically significant (RD = 0.11, 95% CI: −0.20, 0.43; *p* = 0.484). The observed efficacy ranking was: utidelone-induced CIPN > taxane-induced CIPN > CIPN from unspecified agents. Acupuncture also reduced pain intensity (SMD = −0.65, 95% CI: −1.01, −0.29; *p* < 0.001) and FACT-NTX (WMD = 3.66, 95% CI: 1.00, 6.32; *p* = 0.007). No significant differences were found for peroneal nerve conduction velocity (WMD = 1.07, 95% CI: −4.25, 6.39; *p* = 0.694), quality of life score (SMD = 0.54, 95% CI: −0.20, 1.27; *p* = 0.153), or incidence of adverse reactions (RD = 0.03, 95% CI: −0.07, 0.13; *p* = 0.540).

**Conclusion:**

In breast cancer patients with CIPN, acupuncture improved clinical efficacy, reduced pain intensity, and enhanced FACT-NTX scores, particularly in utidelone- and taxane-related cases. No clear benefits were seen for nerve conduction velocity, quality of life score, or incidence of adverse reactions. These findings support acupuncture as a safe and effective adjunct for CIPN symptom management in breast cancer patients.

**Systematic review registration:**

https://www.crd.york.ac.uk/, identifier [CRD42024615214].

## 1 Introduction

Chemotherapy is a crucial component of breast cancer treatment, particularly in adjuvant and neoadjuvant settings (1). However, chemotherapy-induced peripheral neuropathy (CIPN) is a common side effect, characterized by pain, numbness, and sensory abnormalities, which significantly impair quality of life (2). Studies have shown that commonly used chemotherapeutic drugs such as taxanes, platinum compounds, vinca alkaloids, and fluorouracil can induce neuropathy with incidence rates ranging from 57 to 100%, and 11 to 80% of survivors may continue to experience CIPN symptoms 1–3 years after chemotherapy, with 36.5% of patients still suffering from CIPN 5 years post-chemotherapy (3, 4). Short-term symptoms often necessitate chemotherapy dose adjustments or discontinuation, significantly impacting cancer treatment progression (5, 6). Moreover, CIPN can lead to psychological burdens, sleep disturbances, anxiety, and depression in patients (7, 8). Therefore, controlling CIPN symptoms is essential for improving the quality of life in breast cancer patients.

To date, many drugs used for CIPN treatment have shown unsatisfactory efficacy. Acupuncture, as a traditional Chinese medical therapy, has demonstrated significant efficacy in improving peripheral neuropathy with no apparent side effects and has been proven effective in treating various diseases causing peripheral neuropathy (9, 10). However, there is no consensus on the effectiveness and safety of acupuncture in treating breast cancer CIPN. This study aims to conduct a systematic evaluation and meta-analysis of the effectiveness and safety of acupuncture in treating breast cancer CIPN, providing evidence-based medical evidence for its clinical application.

## 2 Methods

This systematic review strictly adhered to the Preferred Reporting Items for Systematic Reviews and Meta-Analyses (PRISMA) guidelines (11) and followed methodological standards outlined in the Cochrane Handbook for Systematic Reviews of Interventions (12). The protocol was registered with the International Prospective Register of Systematic Reviews (PROSPERO), with the assigned registration identifier CRD42024615214.

### 2.1 Literature search

A systematic search was conducted across multiple databases, including PubMed, Embase, Web of Science, Cochrane Library, China National Knowledge Infrastructure, Wanfang Database, VIP Database, and Chinese Biomedical Literature Database. The search period extended from the inception of each database to August 3, 2025. A comprehensive search strategy was developed using a combination of mesh terms, keywords, and text words related to breast cancer, peripheral nervous system diseases, acupuncture, and randomized controlled trials. Boolean operators were employed to ensure thorough coverage. The complete search strategy for each database is detailed in Supplementary material 1.

### 2.2 Eligibility criteria

The inclusion criteria for the articles were as follows: (1) Participants: breast cancer patients diagnosed according to the National Comprehensive Cancer Network clinical practice guidelines for breast cancer, who developed CIPN of grade I or above (13); (2) Intervention measures: the treatment group received acupuncture treatment, including electroacupuncture, manual acupuncture, or other standardized acupuncture modalities; (3) Control measures: the control group received conventional western medicine, routine care, or sham acupuncture treatment; (4) Primary outcome measure: clinical efficacy. Secondary outcome measures: pain intensity, FACT-NTX, nerve conduction velocity, quality of life score and incidence of adverse reactions; (5) Study type: randomized controlled trials (RCTs). Articles meeting any of the following conditions were excluded: (1) Reviews or protocols; (2) Fundamental research; (3) Control group included acupuncture; (4) Incomplete data descriptions.

### 2.3 Study selection

Two reviewers independently screened the titles and abstracts of the retrieved studies to identify potentially relevant studies that met the inclusion criteria. Subsequently, the full texts of the potentially relevant studies were independently reviewed by the same two reviewers to select studies suitable for inclusion in this review. In case of disagreement or uncertainty regarding the inclusion of studies, consensus was reached through consultation with a third reviewer.

### 2.4 Data extraction

The extracted critical information included the following aspects: basic information (authors, publication year, country, participant age, and participant gender), intervention details (type, duration, frequency and stimulation time), comparison measures, outcome measures and sample size. Two reviewers independently extracted data, and in case of any disagreements in data extraction, they were resolved through discussion between the two reviewers and consultation with a third reviewer to reach consensus. For missing data, we contacted the corresponding authors of the relevant studies via email to obtain the required information.

### 2.5 Risk of bias assessment

The risk of bias in the included studies was assessed using the Cochrane risk of bias tool ROB 2.0 (14), which evaluates the following seven aspects: random sequence generation, allocation concealment, blinding of participants and personnel, blinding of outcome assessment, incomplete outcome data, selective reporting and other biases. Each aspect was evaluated as “low risk,” “high risk,” or “unclear risk.” RevMan 5.2 was used to generate the risk of bias figure.

### 2.6 Statistical analysis

We used RevMan 5.2 and Stata 16.0 for meta-analysis. For binary variables, we used risk difference (RD) and 95% confidence interval (CI). For continuous outcomes, the pooled effect size was calculated as the weighted mean difference (WMD) or the standardized mean difference (SMD), each presented with their corresponding 95% CI, depending on whether all studies used the same measurement scale or not. We used the *I*^2^ test to assess heterogeneity. If the heterogeneity test result was *I*^2^ < 50% and *p* > 0.1, we used a fixed-effects model; otherwise, we used a random-effects model. We used Egger’s test and Begg’s test to assess publication bias. To ensure the robustness of the results, we performed sensitivity analysis.

## 3 Results

### 3.1 Literature screening

**Figure 1:**
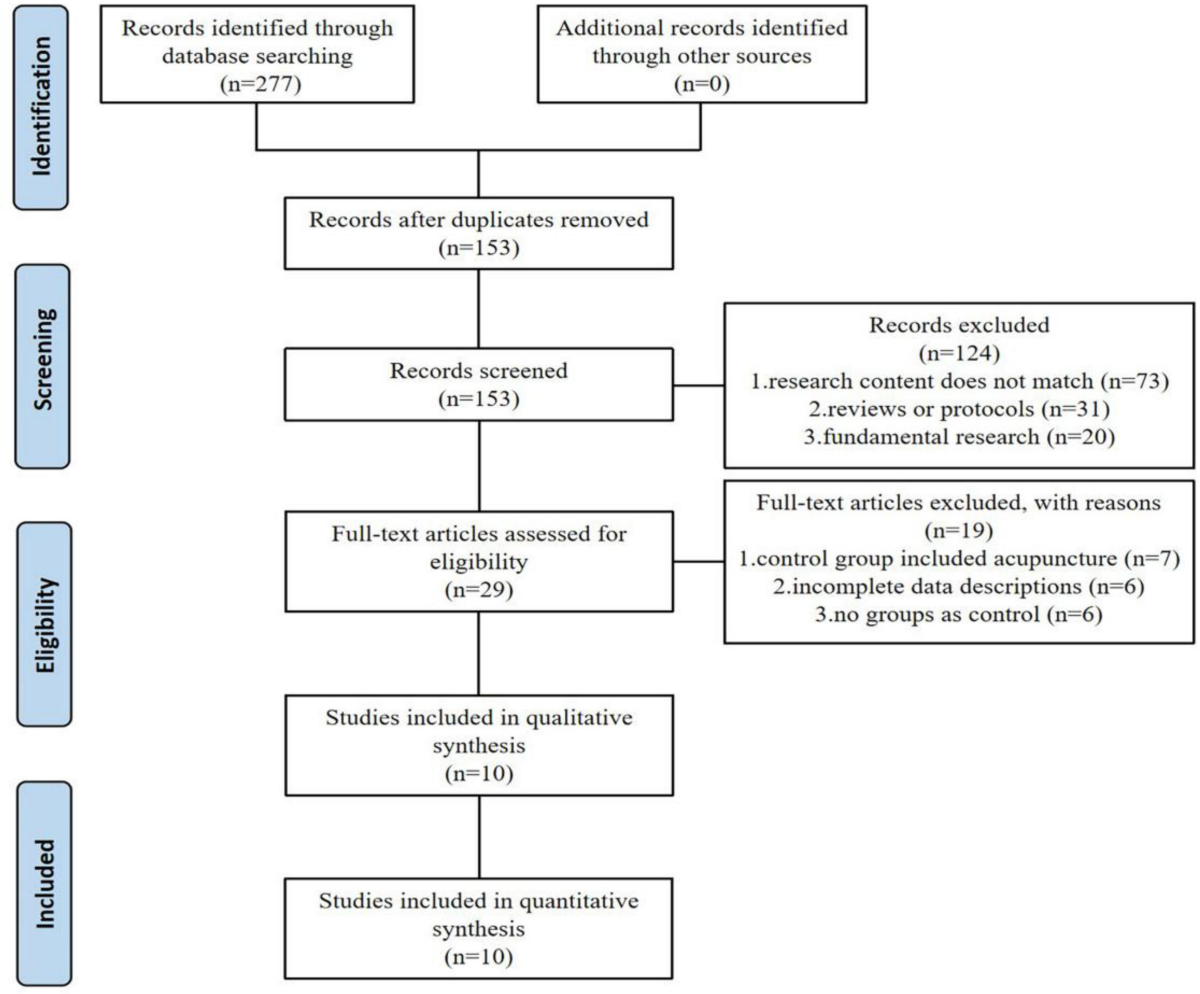
Flow diagram of the selection process of literature.

A comprehensive literature search identified 277 records through electronic database retrieval. After removal of duplicates, 153 unique records were retained for screening, of which 124 were excluded following title and abstract assessment. The remaining 29 full-text articles were evaluated for eligibility, and 19 were excluded after detailed review. Ultimately, 10 studies satisfied the inclusion criteria and were incorporated into the subsequent meta-analysis (Figure 1).

### 3.2 Basic characteristics of included studies

A total of 10 RCTs were included, all published in Chinese or English, and spanning the period 2016–2025. These articles involved a total of 653 patients, with 363 in the acupuncture group and 290 in the control group. Studies were conducted predominantly in China (8 trials), with one trial each from the United States and India. Treatment duration ranged from 20 days to 9 weeks, treatment frequency among included studies ranged from 1 session per week to once daily (with a plurality of trials employing 3 sessions per week), and per-session duration was typically 25–30 min. The basic characteristics of the included studies are shown in Table 1.

**Table 1:** Basic characteristics of the literature included.

Author (year)	Country	Sample size (T/C)	Gender	Age (T/C)	Treatment group				Control group	Outcomes
					Type	Duration	Frequency	Stimulation time		
Bajpai J 2024, (15)	India	52/52	Female	50 (43–57)	AC + UC	6 weeks	2 sessions/week (CIPN grade 1) 3 sessions/week (CIPN grade ≥2)	NR	UC	②⑤
Huang CC 2021, (16)	China	10/10	Female	47.10 ± 11.07/52.10 ± 11.18	AC	9 weeks	2 sessions/week (Weeks 1–6) 1 session/week (Weeks 7–9)	30 min	Sham-AC	②⑥
Li CL 2023, (17)	China	29/29	Female	56.90 ± 6.30/57.10 ± 5.90	EA + BLT	20 days	Once daily	30 min	ME	①②④⑥
Li YM 2025, (18)	China	30/30	Female	51.6 ± 2.7/50.2 ± 3.4	AMT + ME	20 days	AMT: Once daily ME: 0.5 mg × 3 times/day	30 min	ME	①
Liu XH 2023, (19)	China	24/24	Female	32.40 ± 2.30/33.90 ± 3.10	WNM + UC	4 weeks	5 sessions/week	30 min	UC	③
Liu XW 2023, (20)	China	30/30	Female	50.23 ± 8.91/52.40 ± 9.97	EA + ME	6 weeks	3 sessions/week	25 min	ME	⑤⑥
Lu C 2024, (21)	China	114/38	Female	53.35 ± 8.62/53.00 ± 10.00	EA	4 weeks	3 sessions/week	30 min	ME	②③⑥
Lu C 2025, (22)	China	30/30	Female	55.40 ± 8.20/53.00 ± 9.41	EA	4 weeks	3 sessions/week	30 min	ME	⑤⑥
Lu WD 2020, (23)	USA	16/17	Female	52.67 ± 10.18/51.88 ± 12.53	AC + EA	8 weeks	3 sessions/week (Weeks 1–2) 2 sessions/week (Weeks 3–8)	30 min	UC	②③⑤⑥
Xiong ZF 2016, (24)	China	28/30	Female	58.3 ± 10.4/56.9 ± 10.2	AC	30 days	1 session/3 days	30 min	ME	①④

T, treatment group; C, control group; AC, acupuncture; EA, electroacupuncture; ME, mecobalamin; WNM, warm needling moxibustion; BLT, bloodletting therapy; AMT, acupuncture and moxibustion treatment; UC, usual care; NR, not reported; ① represents the clinical efficacy; ② represents the pain intensity; ③ represents the FACT-NTX; ④ represents the nerve conduction velocity; ⑤ represents the quality of life score; ⑥ represents the incidence of adverse reactions.

### 3.3 Risk of bias assessment

**Figure 2:**
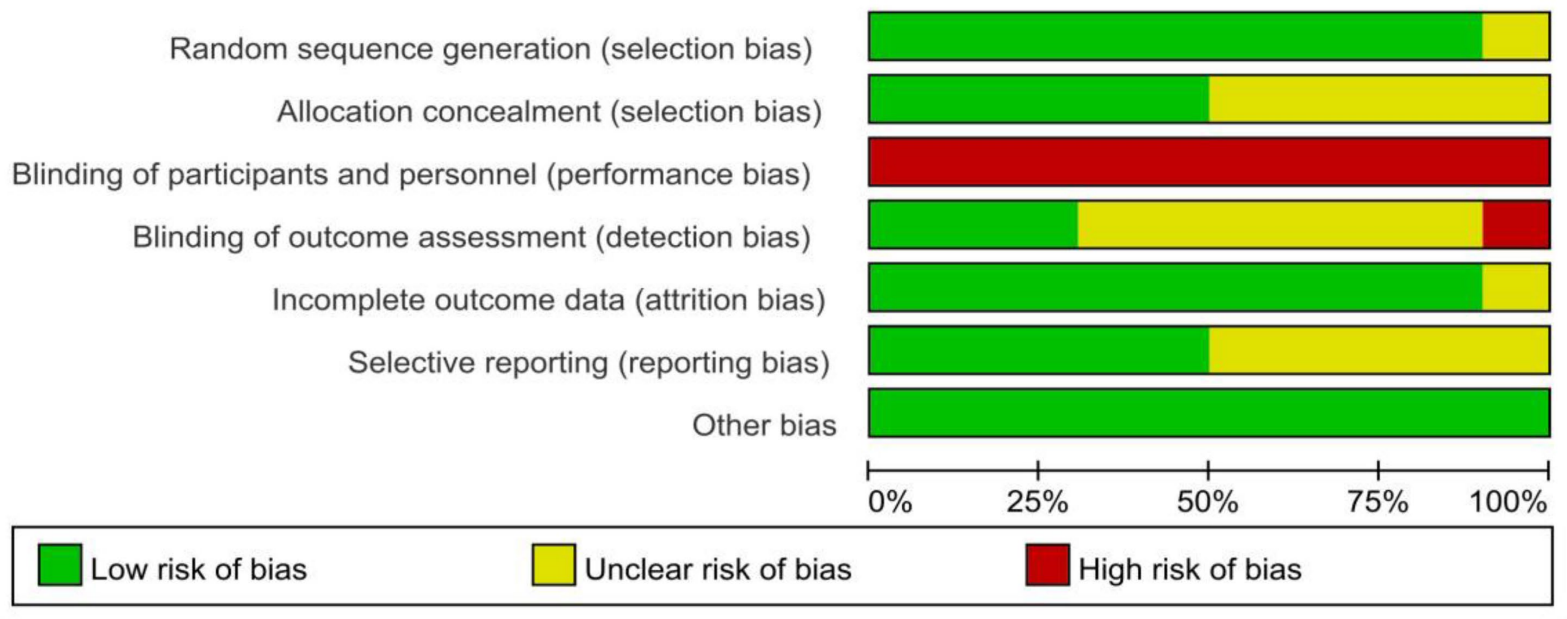
Risk of bias graph for all included studies.

**Figure 3:**
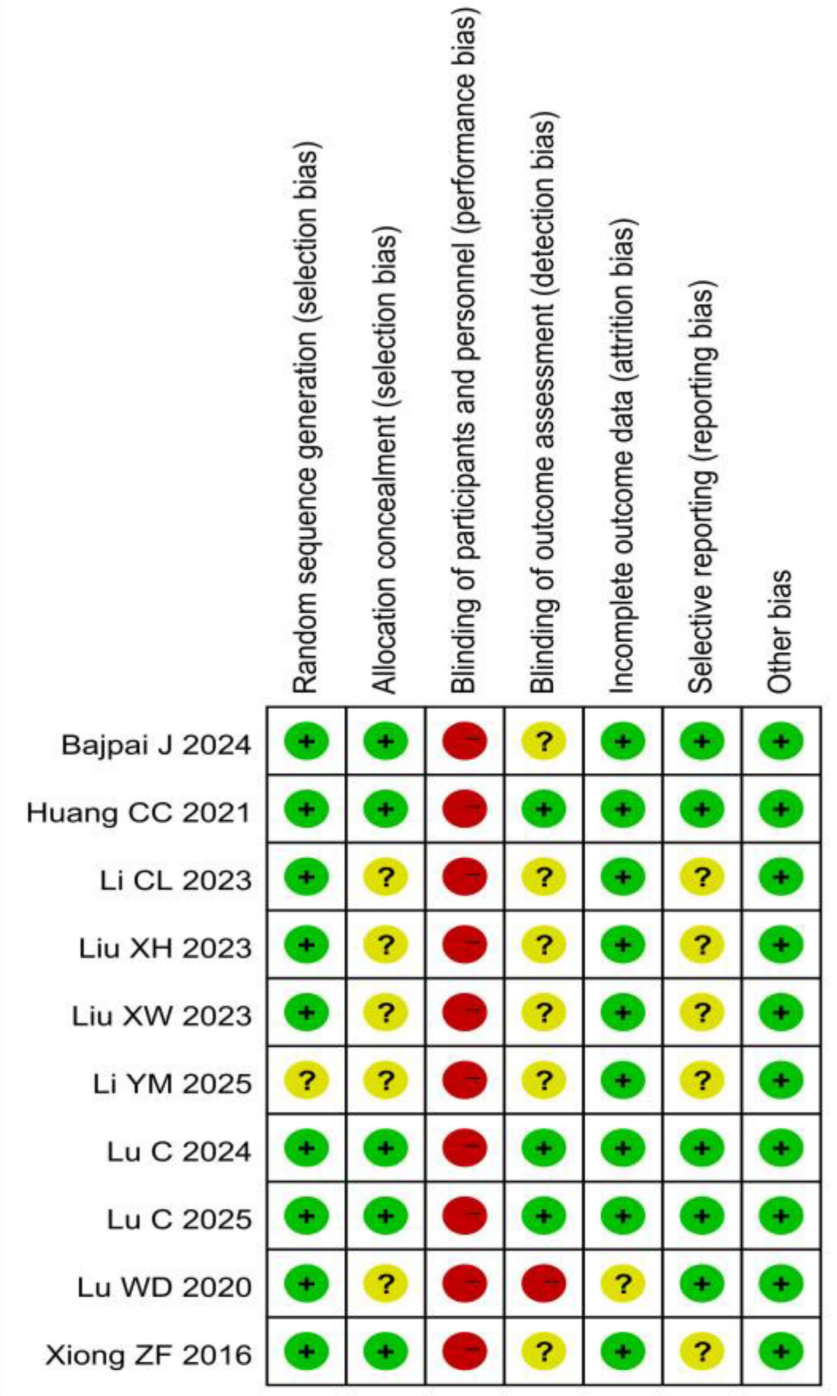
Risk of bias summary for all included studies.

All included studies (15–24) described randomization. Randomization methods included random number tables, computer-generated sequences, and envelope randomization. One study (18) only mentioned “random allocation” without methodological details, warranting an “unclear” risk of bias rating. Allocation concealment was implemented in five studies (15, 16, 21, 22, 24). Due to the nature of the acupuncture intervention, blinding was not feasible for either the researchers or the participants. Blinding of outcome assessors was confirmed in three studies (16, 21, 22). Regarding the completeness of outcome data, four studies (17–20) reported complete data with no dropouts, while five studies (15, 16, 21, 22, 24) showed low attrition rates with balanced reasons for dropout. For selective reporting, five studies (15, 16, 21–23) fully reported all outcomes, while five studies (17–20, 24) lacked sufficient information to assess reporting completeness. No other significant bias sources were identified. The detailed risk of bias is illustrated in Figures 2, 3.

### 3.4 Meta-analysis results

**Figure 4:**
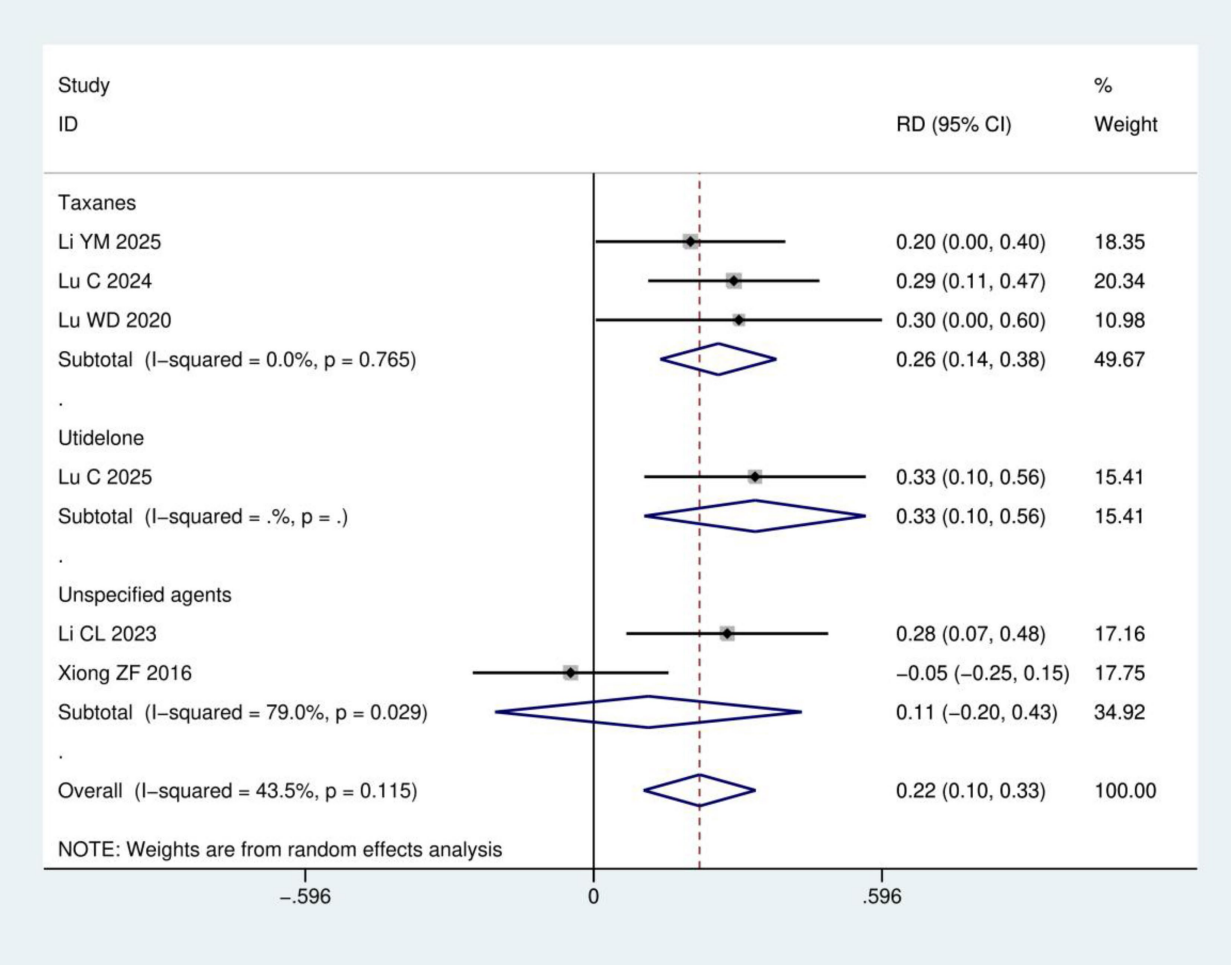
Meta-analysis of clinical efficacy.

Clinical efficacy: A total of six RCTs (17, 18, 21–24) involving 428 patients were included in the clinical efficacy analysis (Figure 4). Based on the chemotherapeutic agents involved, the studies were categorized into three subgroups: taxanes, utidelone, and unspecified agents. Meta-analysis revealed a statistically significant improvement in the clinical efficacy for CIPN following acupuncture treatment group compared to control group (RD = 0.22, 95% CI: 0.10, 0.33; *p* < 0.001). Subgroup analysis according to chemotherapeutic drug class showed that acupuncture was significantly more effective than control for both the taxanes subgroup (3 studies; RD = 0.26, 95% CI: 0.14, 0.38; *p* < 0.001) and the utidelone subgroup (1 study; RD = 0.33, 95% CI: 0.10, 0.56; *p* = 0.004). For the unspecified agents subgroup (2 studies), the result favored acupuncture but did not reach statistical significance (RD = 0.11, 95% CI: −0.20, 0.43; *p* = 0.484). The efficacy ranking from highest to lowest was: utidelone-induced CIPN > taxane-induced CIPN > CIPN from unspecified agents.

**Figure 5:**
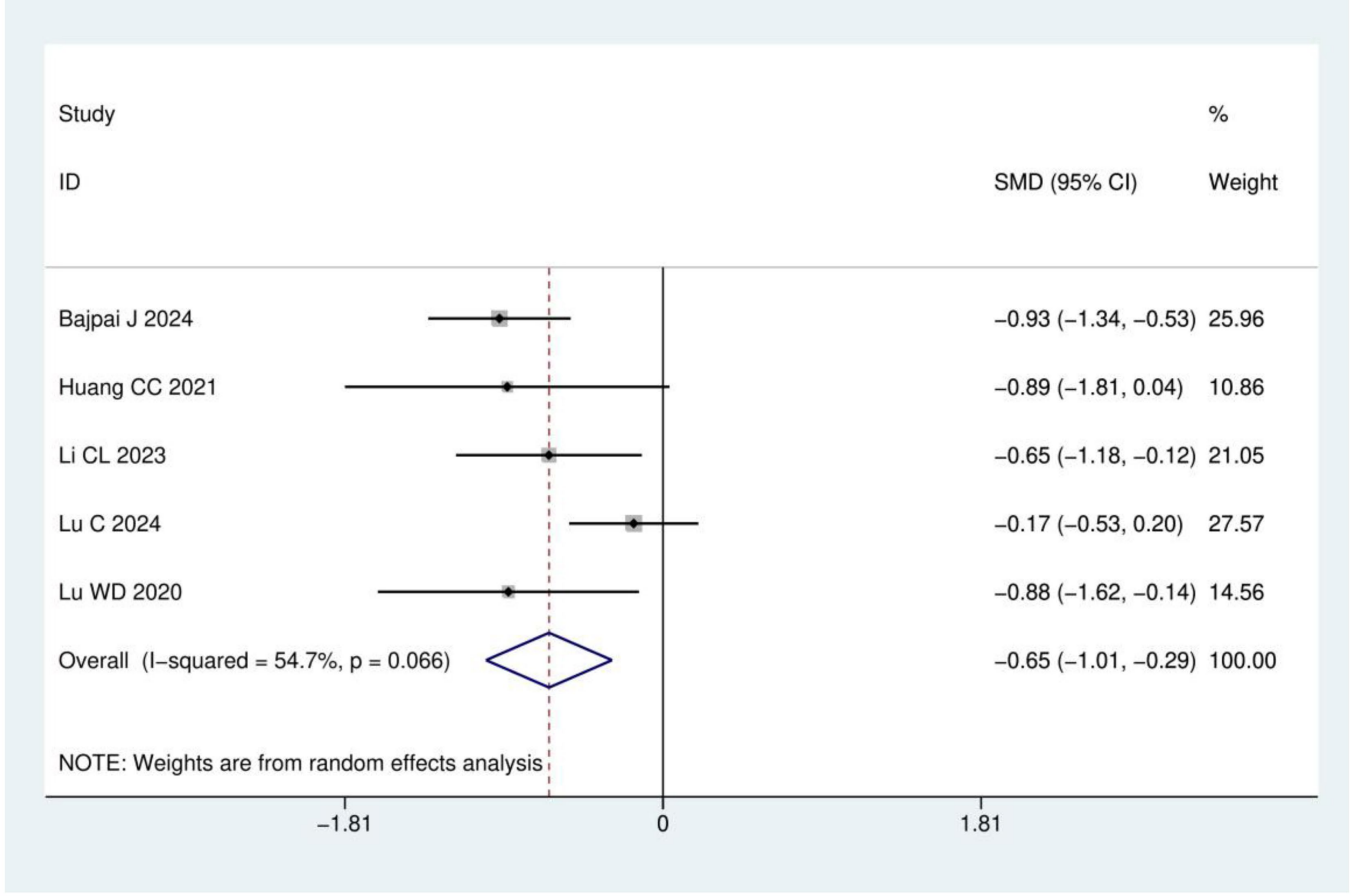
Meta analysis of pain intensity.

Pain intensity: A total of five RCTs (15–17, 21, 23) involving 365 patients were included in the pain intensity analysis. The results of heterogeneity analysis show *I*^2^ = 54.7% and *p* = 0.066. Therefore, a random effects model was used for analysis. Meta-analysis showed that the pain intensity in the acupuncture group was significantly lower than in the control group (SMD = −0.65, 95% CI: −1.01, −0.29; *p* < 0.001), as detailed in Figure 5.

**Figure 6:**
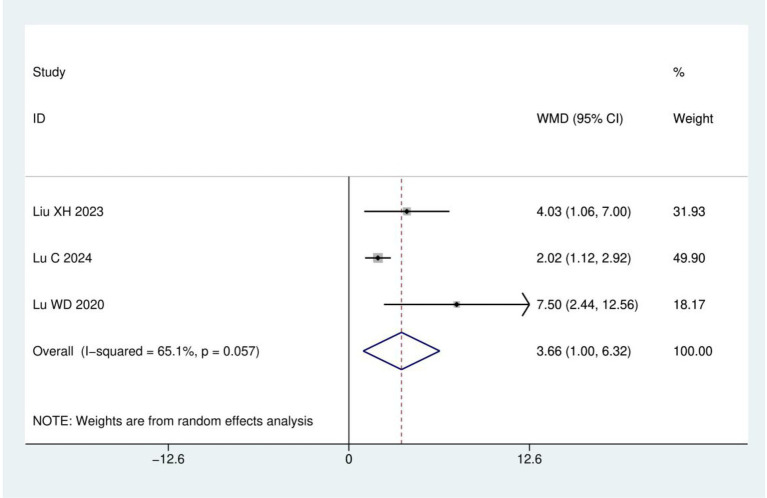
Meta analysis of FACT-NTX.

FACT-NTX: A total of three RCTs (19, 21, 23) were included, involving 233 patients. The results of heterogeneity analysis show *I*^2^ = 65.1% and *p* = 0.057. Therefore, a random effects model was used for analysis. Meta-analysis showed statistically significant difference between the acupuncture and control groups (WMD = 3.66, 95% CI: 1.00, 6.32; *p* = 0.007), as detailed in Figure 6.

**Figure 7:**
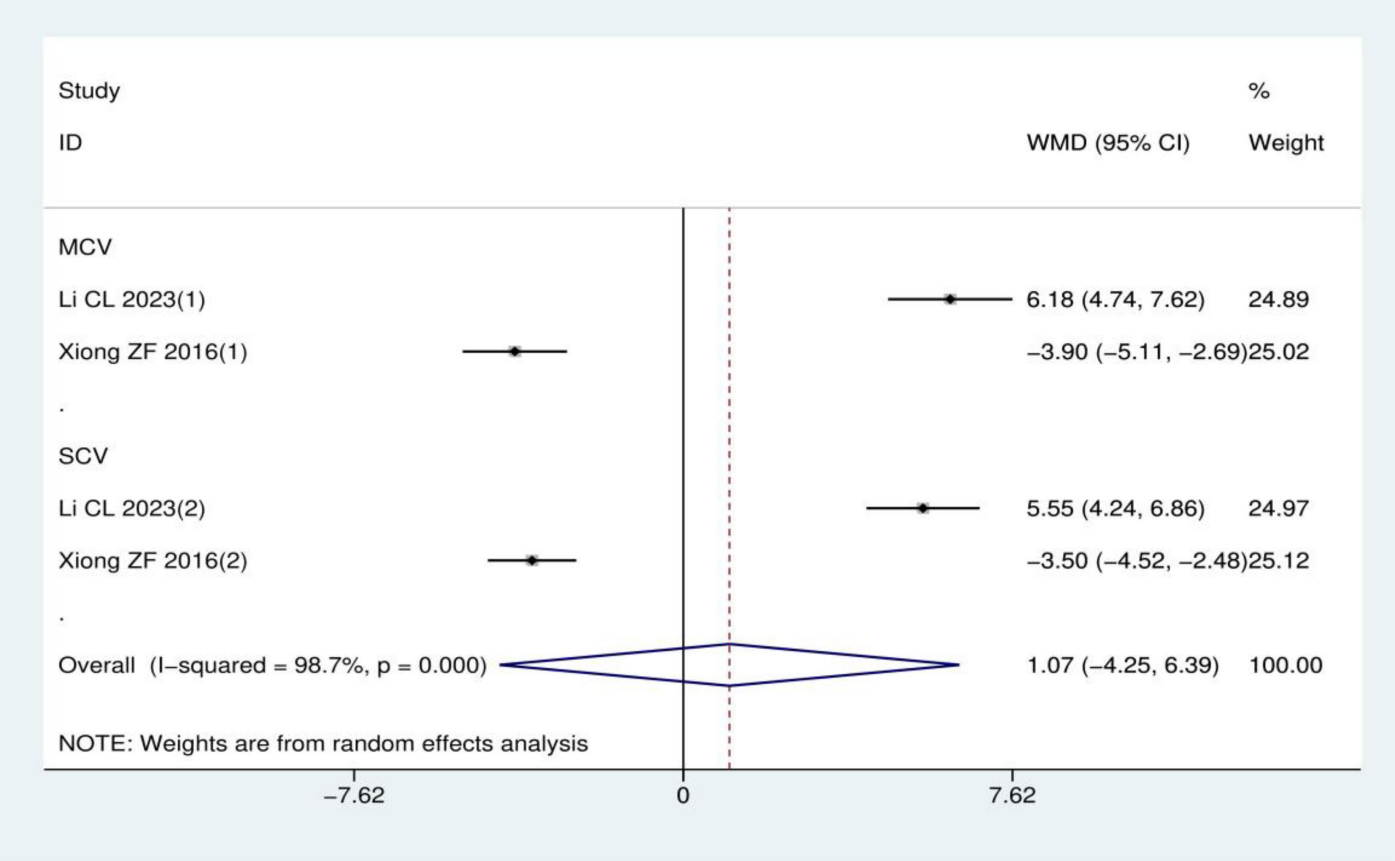
Meta-analysis of peroneal nerve conduction velocity.

Nerve conduction velocity: A total of two RCTs (17, 24) involving 116 patients were included in this analysis, with both studies utilizing acupuncture therapies as the treatment intervention and mecobalamin treatment as the control. Heterogeneity analysis showed *I*^2^ = 98.7% and *p* < 0.001, supporting the use of a random effects model. Notably, the two included studies assessed different nerves besides the peroneal nerve: one measured the median nerve (17), while the other assessed the ulnar nerve (24). Due to this inconsistency in the measured nerves across studies, data could only be pooled for the peroneal nerve. Overall, the meta-analysis demonstrated no statistically significant difference in peroneal nerve conduction velocity between acupuncture therapies and mecobalamin treatment (WMD = 1.07, 95% CI: −4.25–6.39; *p* = 0.694), as detailed in Figure 7.

**Figure 8:**
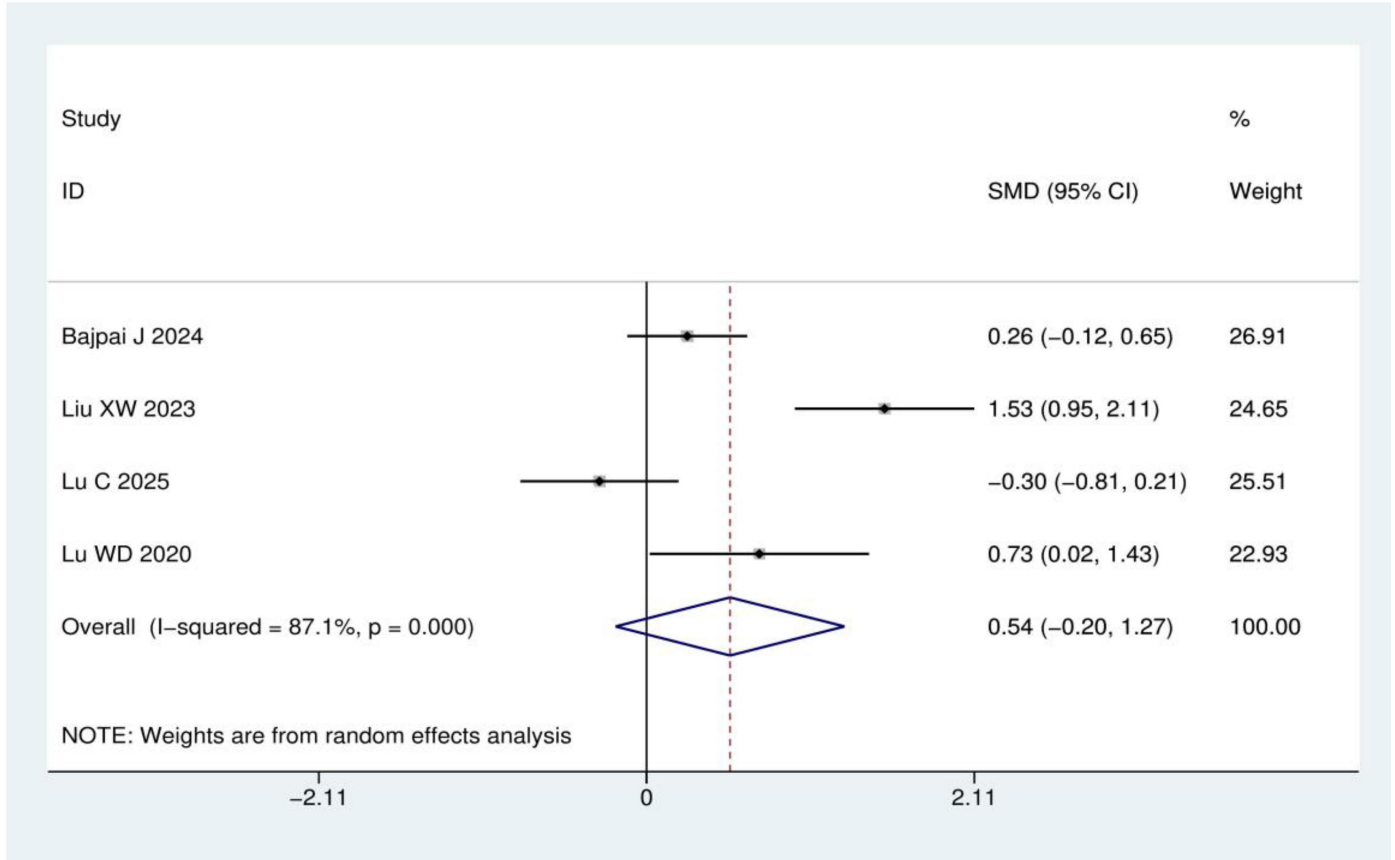
Meta-analysis of quality of life score.

Quality of life score: A total of four RCTs (15, 20, 22, 23) involving 257 patients were included in the quality of life analysis using either the EORTC QLQ-C30 or EORTC QLQ-CIPN20 instruments. Heterogeneity analysis revealed *I*^2^ = 87.1% and *p* < 0.001, leading to the application of a random effects model. Meta-analysis demonstrated no statistically significant difference in quality of life scores between acupuncture group and control group (SMD = 0.54, 95% CI: −0.20, 1.27; *p* = 0.153), as shown in Figure 8.

**Figure 9:**
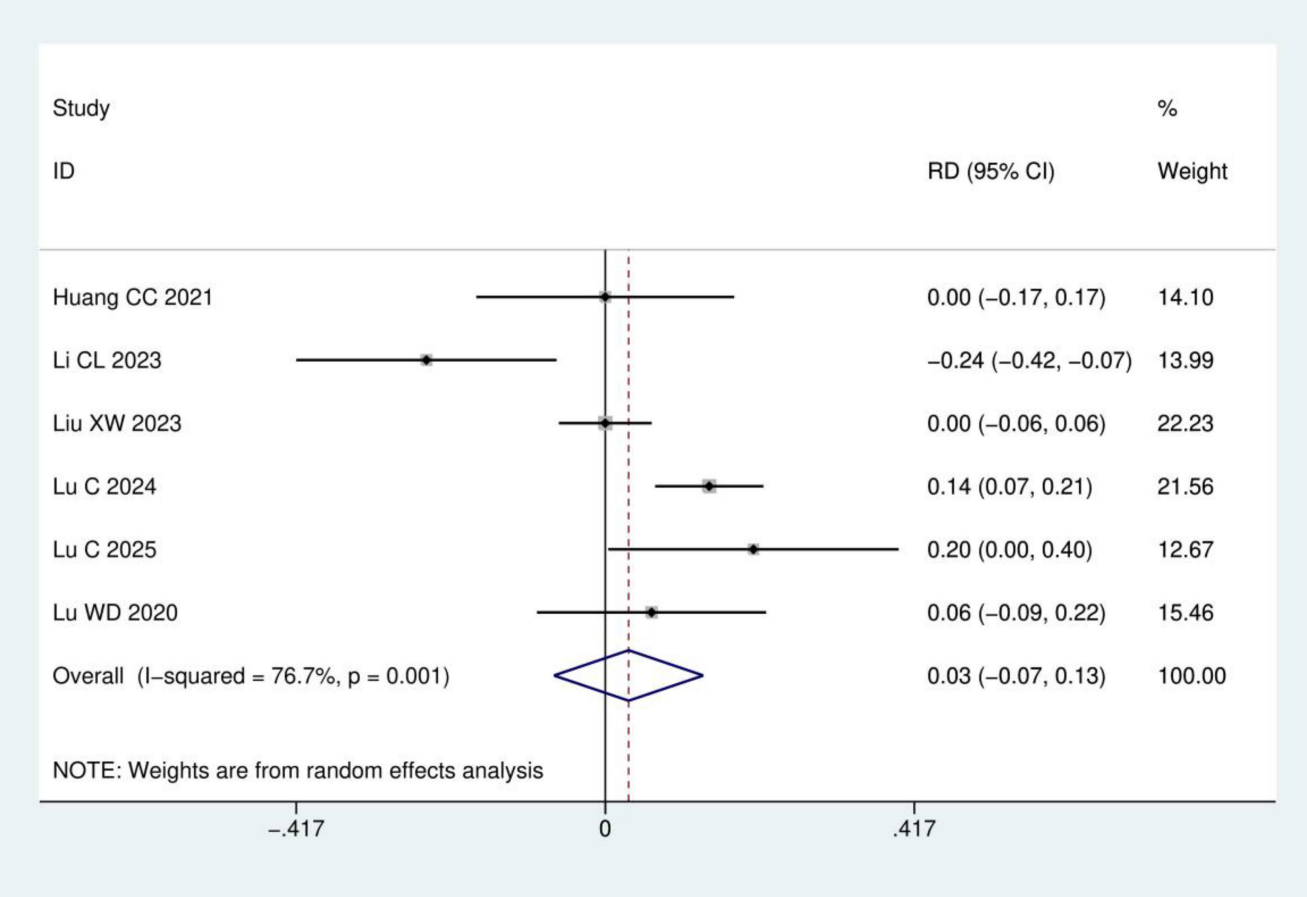
Meta-analysis of incidence of adverse reactions.

Incidence of adverse reactions: A total of six RCTs (16, 17, 20–23) involving 383 patients were included in the analysis of adverse reaction incidence. Heterogeneity analysis indicated significant between-study variation (*I*^2^ = 76.7%, *p* = 0.001). Therefore, a random-effects model was employed. Meta-analysis demonstrated no statistically significant difference in adverse reaction incidence between acupuncture group and control group (RD = 0.03, 95% CI: −0.07, 0.13; *p* = 0.540), as detailed in Figure 9.

### 3.5 Sensitivity analysis

**Figure 10:**
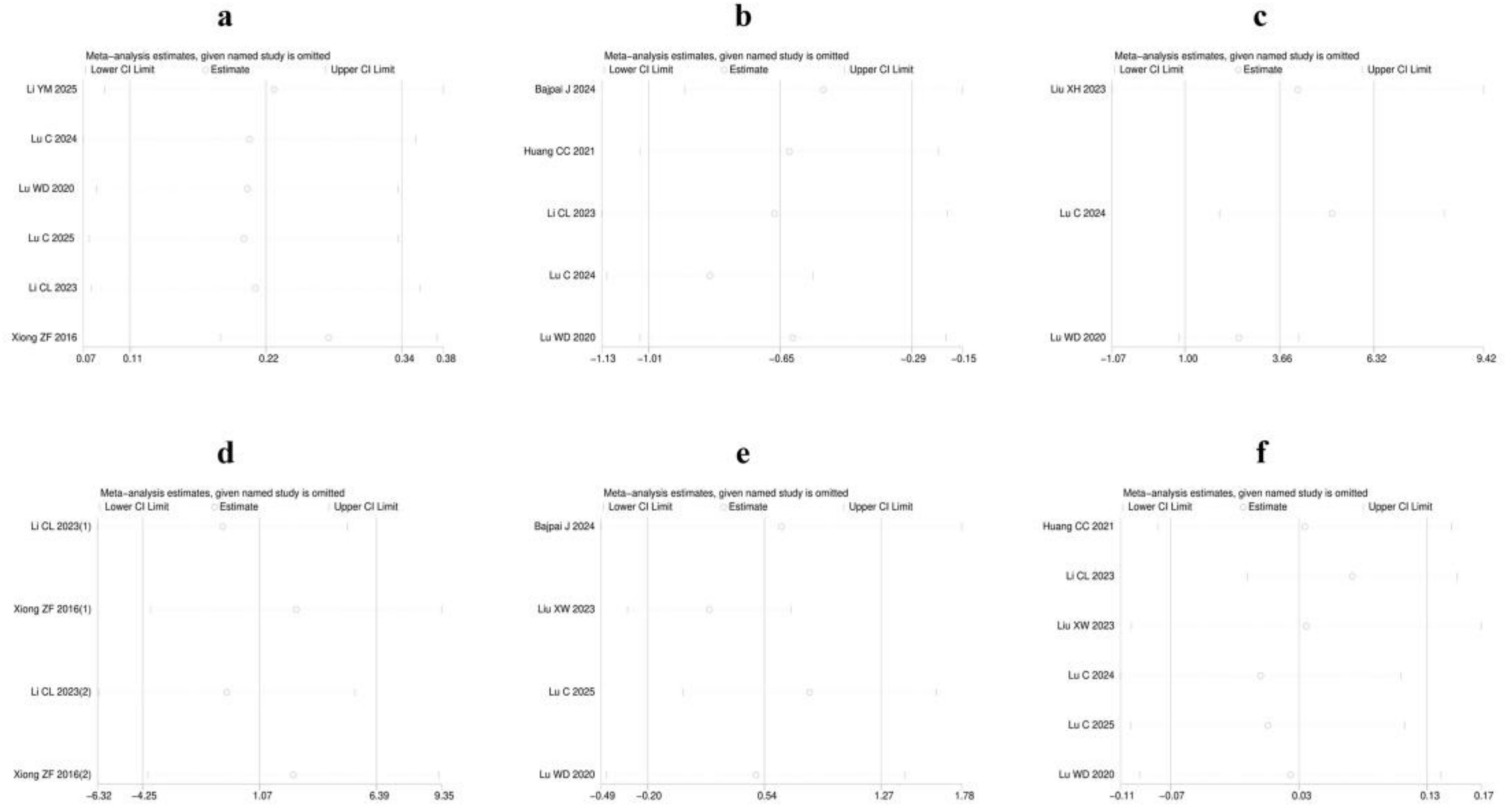
Sensitivity analysis: **(a)** Clinical efficacy; **(b)** Pain intensity; **(c)** FACT-NTX; **(d)** Nerve conduction velocity; **(e)** Quality of life score; **(f)** Incidence of adverse reactions.

Sensitivity analyses were conducted for each of the six outcome measures: clinical efficacy, pain intensity, FACT-NTX, nerve conduction velocity, quality of life score and incidence of adverse reactions. These analyses consistently demonstrated that the effect estimates remained robust, with no variations exceeding the boundaries of the Lower Cl Limit or the Upper Cl Limit. Consequently, the findings of the present study are considered highly robust, as detailed in Figure 10.

### 3.6 Publication bias assessment

Publication bias was assessed for all six outcome measures (clinical efficacy, pain intensity, FACT-NTX scores, nerve conduction velocity, quality of life scores and incidence of adverse reactions) using Egger’s test and Begg’s test (Supplementary material 2). The *p*-values for both tests exceeded 0.05 across all outcome measures, indicating no significant publication bias was present in the current meta-analysis.

## 4 Discussion

Chemotherapy is a core component of breast cancer treatment, but it often induces peripheral neuropathy, leading to pain, numbness, sensory abnormalities, and motor dysfunction, which significantly reduces quality of life and treatment adherence (25, 26). In recent years, acupuncture has been increasingly utilized as a complementary and alternative therapy for managing malignancy-related symptoms, with growing evidence supporting its efficacy for various chemotherapy-induced sequelae in breast cancer patients. For instance, acupuncture has been shown to significantly improve chemotherapy-associated insomnia, reduce anxiety and depression, and enhance quality of life (27). Furthermore, systematic reviews confirm its effectiveness and safety in alleviating cancer-related fatigue (28, 29), and preclinical studies suggest its actions may involve modulation of the gut-brain axis (30). These findings underscore the potential of acupuncture to address a spectrum of neuropsychiatric and somatic side effects. Acupuncture has also demonstrated promise in alleviating peripheral neuropathy caused by various etiologies, including improvements in pain intensity and nerve conduction velocity (31, 32). However, no comprehensive investigation has systematically examined the therapeutic efficacy of acupuncture specifically for CIPN in breast cancer patients. This critical evidence gap warrants urgent addressing. To provide a broader perspective, we summarize ongoing or recently completed phase III RCTs of acupuncture for CIPN, including official title, trial phase, expected completion date, current status, and corresponding references or registration links (Table 2).

**Table 2:** Phase III RCTs of acupuncture for CIPN.

Official title	Trial phase	Expected completion date	Current status	Corresponding references or registration links
A randomized phase III clinical trial of acupuncture for chemotherapy-induced peripheral neuropathy treatment (ACT)	Phase III	Estimated study completion: April 2026	Recruiting	https://clinicaltrials.gov/study/NCT04917796
Acupuncture as a modality of treatment for chemotherapy-induced peripheral neuropathy in breast cancer: A phase 3 randomized controlled trial (ABC-CIPN)	Phase III	Study completion: 30 April 2024	Completed	DOI: 10.1200/JCO.2024.42.16_suppl.12126

This meta-analysis incorporated 10 RCTs involving 653 patients, demonstrating that acupuncture significantly improves pain and neurotoxicity-specific symptoms compared to control interventions in breast cancer patients with CIPN, while maintaining a comparable safety profile. These findings support the use of acupuncture as a valuable complementary therapy for symptomatic management in this patient population. When compared specifically to mecobalamin, acupuncture shows similar effects on peroneal nerve conduction velocity. Only two trials contributed nerve conduction data, resulting in small sample size and very high statistical heterogeneity (*I*^2^ = 98.7%). This substantial heterogeneity and limited number of trials reduce power to detect a true effect on conduction velocity and increase uncertainty of the pooled estimate. The direct comparisons for nerve conduction velocity were against mecobalamin. When acupuncture is compared with an active pharmacologic comparator that may have direct effects on nerve physiology, a lack of statistical superiority in conduction velocity does not contradict clinically meaningful symptomatic improvement.

Acupuncture may alleviate CIPN in breast cancer patients through a multifaceted interplay of neuroimmune regulation, inflammatory cytokine modulation, microcirculatory enhancement, and upregulation of neurotrophic factors. Neuroimmune crosstalk plays a pivotal role in CIPN pathogenesis, wherein chemotherapy triggers infiltration of macrophages and activation of spinal microglia, leading to peripheral and central sensitization (33–35). Acupuncture has been shown to inhibit microglial activation and shift macrophage polarization from the pro-inflammatory M1 to the anti-inflammatory M2 phenotype (36, 37), thereby reducing the release of pro-inflammatory cytokines such as TNF-*α*, IL-1β, and IL-6 (38, 39). In addition to immune modulation, acupuncture improves microcirculation, which is often compromised in CIPN. Studies have demonstrated that acupuncture increases local blood flow in both skin and muscle tissues, likely through somato-autonomic reflexes that modulate sympathetic and parasympathetic tone (40, 41). Enhanced microcirculation may facilitate nutrient delivery and waste removal, thereby supporting nerve repair and reducing ischemic damage to peripheral nerves. Furthermore, acupuncture has been shown to regulate neurotrophic factors, particularly brain-derived neurotrophic factor (BDNF), which is critical for neuronal survival, axonal regeneration, and synaptic plasticity (42). In various neuropathy models, acupuncture upregulated BDNF expression and activated downstream signaling pathways such as PI3K/Akt and MAPK/ERK, promoting neuroprotection and functional recovery (43, 44).

Our study has several limitations. First, the included clinical trials were all single-center designs, and most of them had small sample sizes, which may limit the generalizability of the results. Second, there was a lack of long-term follow-up data to assess the sustained effects of acupuncture on the long-term efficacy of breast cancer CIPN. Third, differences in acupuncture parameters (such as acupoints, stimulation frequency, and duration) across studies may have contributed to heterogeneity. Future research should optimize study design, such as conducting multi-center, large-sample RCTs and follow-up assessments to confirm the long-term efficacy of acupuncture. Additionally, using standardized acupuncture protocols (such as evidence-based acupuncture parameter selection) would enhance consistency across studies. To enable biomarker-guided selection for acupuncture in the prevention and treatment of CIPN, future studies should integrate baseline clinical phenotypes (symptom pattern, CIPN duration, chemotherapy class and cumulative dose) with objective molecular and neurophysiological markers to build and externally validate predictive models. Prospective, biomarker-enriched and stratified RCTs with pre-specified subgroup analyses and harmonized endpoints are therefore warranted to identify patients most likely to derive preventive or therapeutic benefit from acupuncture.

## 5 Conclusion

In breast cancer patients with CIPN, acupuncture enhanced clinical efficacy and improved pain intensity and FACT-NTX scores, especially for utidelone- and taxane-induced neuropathy. No significant effects were observed on nerve conduction velocity, quality of life score, or incidence of adverse reactions. Overall, acupuncture is a safe and effective adjunctive therapy for CIPN in breast cancer patients, warranting further study to clarify its impact on objective outcomes.

## Data Availability

The original contributions presented in the study are included in the article/Supplementary material, further inquiries can be directed to the corresponding author/s.

## References

[1] Tamirisa N Hunt KK. Neoadjuvant chemotherapy, endocrine therapy, and targeted therapy for breast cancer: ASCO guideline. Ann Surg Oncol. (2022) 29:1489–92. doi: 10.1245/s10434-021-11223-3, 34989940

[2] Loprinzi CL Lacchetti C Bleeker J Cavaletti G Chauhan C Hertz DL . Prevention and management of chemotherapy-induced peripheral neuropathy in survivors of adult cancers: ASCO guideline update. J Clin Oncol. (2020) 38:3325–48. doi: 10.1200/JCO.20.01399, 32663120

[3] Ma J Kavelaars A Dougherty PM Heijnen CJ. Beyond symptomatic relief for chemotherapy-induced peripheral neuropathy: targeting the source. Cancer. (2018) 124:2289–98. doi: 10.1002/cncr.31248, 29461625 PMC5991994

[4] Desforges AD Hebert CM Spence AL Reid B Dhaibar HA Cruz-Topete D . Treatment and diagnosis of chemotherapy-induced peripheral neuropathy: an update. Biomed Pharmacother. (2022) 147:112671. doi: 10.1016/j.biopha.2022.112671, 35104697 PMC11118018

[5] Colvin LA. Chemotherapy-induced peripheral neuropathy: where are we now? Pain. (2019) 160 Suppl 1:S1–S10. doi: 10.1097/j.pain.0000000000001540, 31008843 PMC6499732

[6] Burgess J Ferdousi M Gosal D Boon C Matsumoto K Marshall A . Chemotherapy-induced peripheral neuropathy: epidemiology, pathomechanisms and treatment. Oncol Ther. (2021) 9:385–450. doi: 10.1007/s40487-021-00168-y, 34655433 PMC8593126

[7] Kerckhove N Collin A Condé S Chaleteix C Pezet D Balayssac D. Long-term effects, pathophysiological mechanisms, and risk factors of chemotherapy-induced peripheral neuropathies: a comprehensive literature review. Front Pharmacol. (2017) 8:86. doi: 10.3389/fphar.2017.00086, 28286483 PMC5323411

[8] Tofthagen CS Cheville AL Loprinzi CL. The physical consequences of chemotherapy-induced peripheral neuropathy. Curr Oncol Rep. (2020) 22:50. doi: 10.1007/s11912-020-00903-0, 32323068

[9] Feng Z Cui S Yang H Wang Y Zhou X Wong J . Acupuncture for neuropathic pain: a meta-analysis of randomized control trials. Front Neurol. (2022) 13:1076993. doi: 10.3389/fneur.2022.1076993, 36698895 PMC9868276

[10] Liu J Lin Y Huang Y Yang Q Li X Ye Y . Efficacy and safety of acupuncture for painful diabetic neuropathy: a systematic review and meta-analysis. Front Neurol. (2024) 15:1402458. doi: 10.3389/fneur.2024.1402458, 38903165 PMC11188462

[11] Page MJ McKenzie JE Bossuyt PM Boutron I Hoffmann TC Mulrow CD . The PRISMA 2020 statement: an updated guideline for reporting systematic reviews. BMJ. (2021) 372:n71. doi: 10.1136/bmj.n71, 33782057 PMC8005924

[12] Cumpston M Li T Page MJ Chandler J Welch VA Higgins JP . Updated guidance for trusted systematic reviews: a new edition of the Cochrane handbook for systematic reviews of interventions. Cochrane Database Syst Rev. (2019) 10:ED000142. doi: 10.1002/14651858.ED000142, 31643080 PMC10284251

[13] Gradishar WJ Moran MS Abraham J Abramson V Aft R Agnese D . Breast cancer, version 3.2024, NCCN clinical practice guidelines in oncology. J Natl Compr Cancer Netw. (2024) 22:331–57. doi: 10.6004/jnccn.2024.0035, 39019058

[14] Sterne JAC Savović J Page MJ Elbers RG Blencowe NS Boutron I . RoB 2: a revised tool for assessing risk of bias in randomised trials. BMJ. (2019) 366:l4898. doi: 10.1136/bmj.l4898 31462531

[15] Bajpai J Kapu V Modi J Rath S Pawar A Siddiqui A . Acupuncture as a modality of treatment for chemotherapy-induced peripheral neuropathy in breast cancer: a phase 3 randomized controlled trial (ABC-CIPN) JCO 2024 42 12126 doi: 10.1200/JCO.2024.42.16_suppl.12126

[16] Huang CC Ho TJ Ho HY Chen PY Tu CH Huang YC . Acupuncture relieved chemotherapy-induced peripheral neuropathy in patients with breast Cancer: a pilot randomized sham-controlled trial. J Clin Med. (2021) 10:3694. doi: 10.3390/jcm10163694, 34441990 PMC8397157

[17] Li C Li X Lu Y Bao C Liu Q Liu Y. Clinical study of electroacupuncture combined with venesection in treating chemotherapy-induced peripheral neuropathy in breast cancer patients. Mod Chin Med. (2023) 43:65–9. doi: 10.13424/j.cnki.mtcm.2023.04.013

[18] Li Y Zhai Y Hao X Zhang Y Ma X. Clinical study of abdominal acupuncture combined with moxibustion for the treatment of peripheral neurotoxicity due to paclitaxel-based chemotherapeutic agents in breast cancer. Modern J Integr Tradit Chin Western Med. (2025) 34:74–7.

[19] Liu X Zhang Y Huang Y Chao T. Efficacy of finger exercise combined with warm needling in treating chemotherapy-induced peripheral neuropathy in breast cancer patients. Chin J Phys Med Rehabil. (2023) 45:355–7. doi: 10.3760/cma.j.issn.0254-1424.2023.04.015

[20] Liu X. Clinical study of electroacupuncture in the treatment of chemotherapy-induced peripheral neurotoxicity by albumin bound paclitaxel. [master’s thesis]. Jinan: Shandong University of Traditional Chinese Medicine (2023). 61 p.

[21] Lu C Feng X Shen Q Li G Wu T Li X . Electroacupuncture with different frequencies for paclitaxel-induced peripheral neuropathy: a randomized controlled trial. Chin Acupunct Moxibust. (2024) 44:1139–45. doi: 10.13703/j.0255-2930.20240123-0001, 39401811

[22] Lu C Shen Q Deng D Zhang Y Wang P Shao X . Effects of electroacupuncture and mecobalamin for utidelon-induced peripheral neuropathy in breast cancer patients: a randomized controlled clinical trial. JPR. (2025) 18:3593–608. doi: 10.2147/JPR.S526405, 40687335 PMC12276754

[23] Lu W Giobbie-Hurder A Freedman RA Shin IH Lin NU Partridge AH . Acupuncture for chemotherapy-induced peripheral neuropathy in breast cancer survivors: a randomized controlled pilot trial. Oncologist. (2020) 25:310–8. doi: 10.1634/theoncologist.2019-0489, 32297442 PMC7160396

[24] Xiong Z Wang T Gan L Ran J Min J Lü G. Clinical efficacy of acupoint injection for chemotherapy-induced peripheral neuropathy of patients with breast cancer. World J Acupunct Moxibustion. (2016) 26:20–4. doi: 10.1016/S1003-5257(17)30005-3

[25] Montemurro F Mittica G Cagnazzo C Longo V Berchialla P Solinas G . Self-evaluation of adjuvant chemotherapy-related adverse effects by patients with breast Cancer. JAMA Oncol. (2016) 2:445–52. doi: 10.1001/jamaoncol.2015.4720, 26720497

[26] Bao T Basal C Seluzicki C Li SQ Seidman AD Mao JJ. Long-term chemotherapy-induced peripheral neuropathy among breast cancer survivors: prevalence, risk factors, and fall risk. Breast Cancer Res Treat. (2016) 159:327–33. doi: 10.1007/s10549-016-3939-0, 27510185 PMC5509538

[27] Zhang J Qin Z So TH Chang TY Yang S Chen H . Acupuncture for chemotherapy-associated insomnia in breast cancer patients: an assessor-participant blinded, randomized, sham-controlled trial. Breast Cancer Res. (2023) 25:49. doi: 10.1186/s13058-023-01645-0, 37101228 PMC10134666

[28] Choi T-Y Ang L Jun JH Alraek T Birch S Lu W . Acupuncture for managing cancer-related fatigue in breast cancer patients: a systematic review and meta-analysis. Cancer. (2022) 14:4419. doi: 10.3390/cancers14184419, 36139579 PMC9496910

[29] Hu X Feng B Xie J Deng X Zou Y. Is acupuncture an ideal adjunctive treatment for cancer-related fatigue? Comment on Choi et al. acupuncture for managing Cancer-related fatigue in breast Cancer patients: a systematic review and Meta-analysis. Cancers 2022, 14, 4419. Cancer. (2022) 15:223. doi: 10.3390/cancers15010223, 36612219 PMC9818848

[30] Lv Z Liu R Su K Gu Y Fang L Fan Y . Acupuncture ameliorates breast cancer-related fatigue by regulating the gut microbiota-gut-brain axis. Front Endocrinol. (2022) 13:921119. doi: 10.3389/fendo.2022.921119, 36093113 PMC9449876

[31] Kutcher AM LeBaron VT. Evaluating acupuncture for the treatment of chemotherapy-induced peripheral neuropathy: an integrative review. West J Nurs Res. (2022) 44:169–79. doi: 10.1177/0193945921992538, 33559535

[32] Ge R Liu R He M Wu J Zhang F Huang C. The efficacy of acupuncture for diabetic peripheral neuropathy: a systematic review and meta-analysis of randomized controlled trails. Front Neurol. (2024) 15:1500709. doi: 10.3389/fneur.2024.1500709, 39758782 PMC11697586

[33] Fumagalli G Monza L Cavaletti G Rigolio R Meregalli C. Neuroinflammatory process involved in different preclinical models of chemotherapy-induced peripheral neuropathy. Front Immunol. (2021) 11:626687. doi: 10.3389/fimmu.2020.626687, 33613570 PMC7890072

[34] Li Y Xu R Chen M Zheng K Nie H Yin C . Electroacupuncture alleviates paclitaxel-induced peripheral neuropathy by reducing CCL2-mediated macrophage infiltration in sensory ganglia and sciatic nerve. Chin Med. (2025) 20:9. doi: 10.1186/s13020-024-01023-8, 39806462 PMC11727193

[35] Ollodart J Steele LR Romero-Sandoval EA Strowd RE Shiozawa Y. Contributions of neuroimmune interactions to chemotherapy-induced peripheral neuropathy development and its prevention/therapy. Biochem Pharmacol. (2024) 222:116070. doi: 10.1016/j.bcp.2024.116070, 38387528 PMC10964384

[36] Nan F-B Gu Y-X Wang J-L Chen S-D. Electroacupuncture promotes macrophage/microglial M2 polarization and suppresses inflammatory pain through AMPK. Neuroreport. (2024) 35:343–51. doi: 10.1097/WNR.0000000000002005, 38526969

[37] Li X-C Chen H Chen Y Chu Y-X Mi W-L Wang Y-Q . Spinal neuronal miR-124 inhibits microglial activation and contributes to preventive effect of electroacupuncture on chemotherapy-induced peripheral neuropathy in mice. J Immunol. (2024) 212:410–20. doi: 10.4049/jimmunol.2300539, 38088802

[38] Li N Guo Y Gong Y Zhang Y Fan W Yao K . The anti-inflammatory actions and mechanisms of acupuncture from acupoint to target organs via neuro-immune regulation. J Inflamm Res. (2021) 14:7191–224. doi: 10.2147/JIR.S341581, 34992414 PMC8710088

[39] Hung AL Lim M Doshi TL. Targeting cytokines for treatment of neuropathic pain. Scand J Pain. (2017) 17:287–93. doi: 10.1016/j.sjpain.2017.08.002, 29229214 PMC5774983

[40] Takayama S Watanabe M Kusuyama H Nagase S Seki T Nakazawa T . Evaluation of the effects of acupuncture on blood flow in humans with ultrasound color doppler imaging. Evid Based Complement Alternat Med. (2012) 2012:1–8. doi: 10.1155/2012/513638, 22778772 PMC3388479

[41] Kim S-Y Min S Lee H Cheon S Zhang X Park J-Y . Changes of local blood flow in response to acupuncture stimulation: a systematic review. Evid Based Complement Alternat Med. (2016) 2016:9874207. doi: 10.1155/2016/9874207, 27403201 PMC4923553

[42] Xue M Sun Y-L Xia Y-Y Huang Z-H Huang C Xing G-G. Electroacupuncture modulates spinal BDNF/TrκB Signaling pathway and ameliorates the sensitization of dorsal horn WDR neurons in spared nerve injury rats. IJMS. (2020) 21:6524. doi: 10.3390/ijms21186524, 32906633 PMC7555233

[43] Xue X You Y Tao J Ye X Huang J Yang S . Electro-acupuncture at points of Zusanli and Quchi exerts anti-apoptotic effect through the modulation of PI3K/Akt signaling pathway. Neurosci Lett. (2014) 558:14–9. doi: 10.1016/j.neulet.2013.10.029, 24157854

[44] Miao C Li X Zhang Y. Effect of acupuncture on BDNF signaling pathways in several nervous system diseases. Front Neurol. (2023) 14:1248348. doi: 10.3389/fneur.2023.1248348, 37780709 PMC10536971

