# Supplementary figures and images for: Efficacy and safety of acupuncture in the treatment of chemotherapy-induced peripheral neuropathy in breast cancer patients: a systematic review and meta-analysis
Source: Front Neurol. 2026 Jan 14;16:1690446. doi: 10.3389/fneur.2025.1690446 (PMC12847451; doi:10.3389/fneur.2025.1690446)

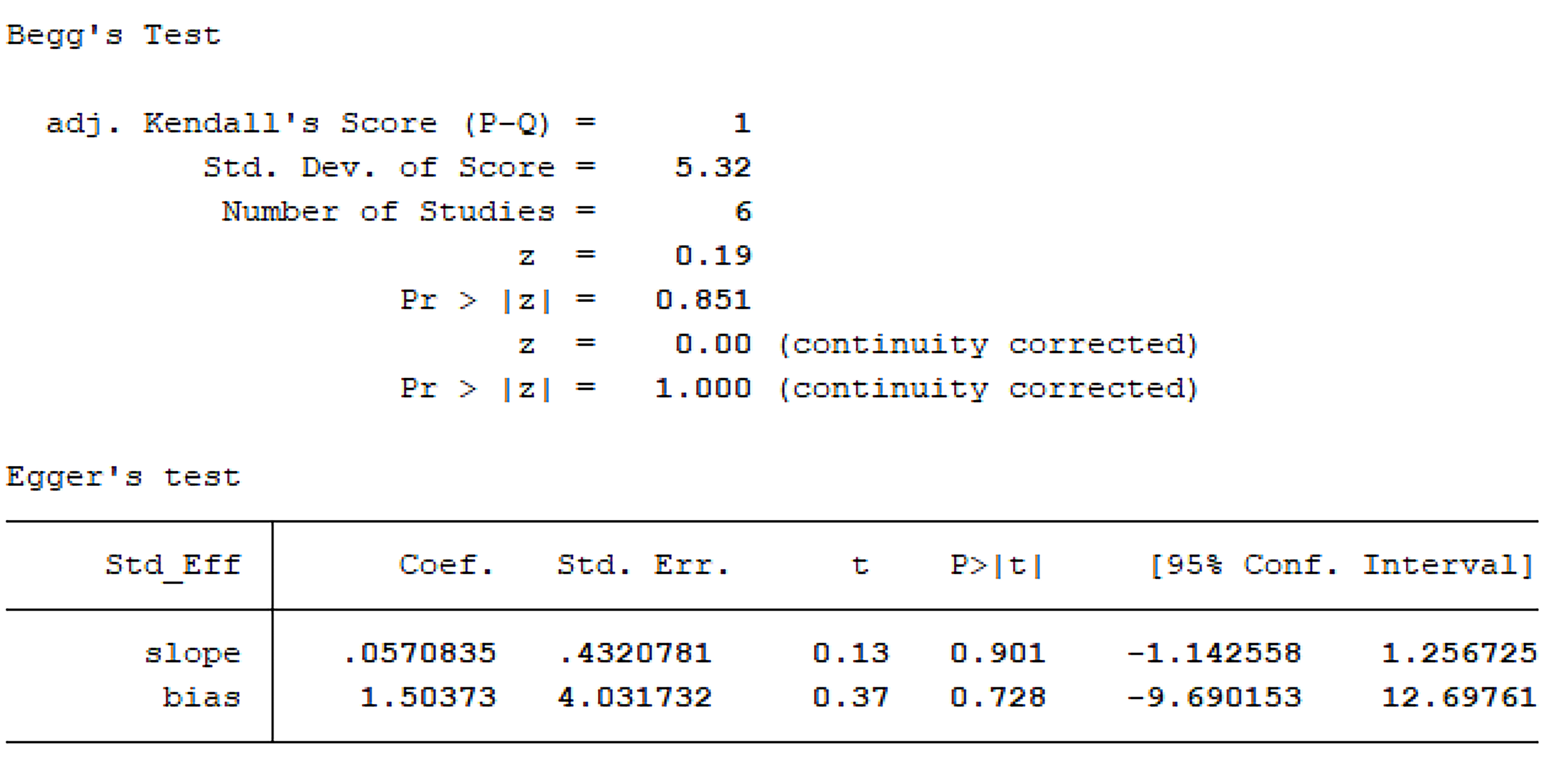

Supplement: Supplementary file 1 [file Data_Sheet_1.zip › Supplementary Material 2/Clinical efficacy.jpg]

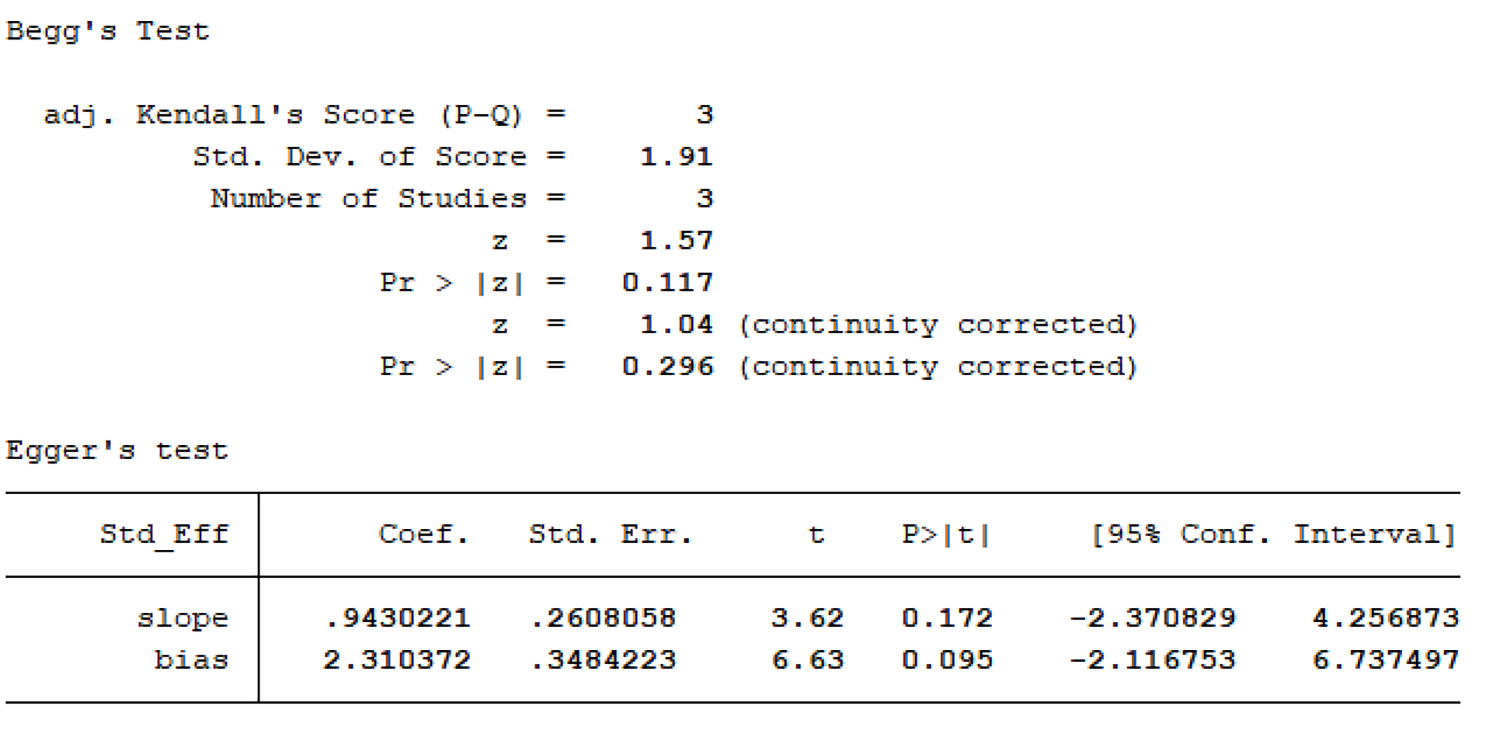

Supplement: Supplementary file 1 [file Data_Sheet_1.zip › Supplementary Material 2/FACT-NTX.jpg]

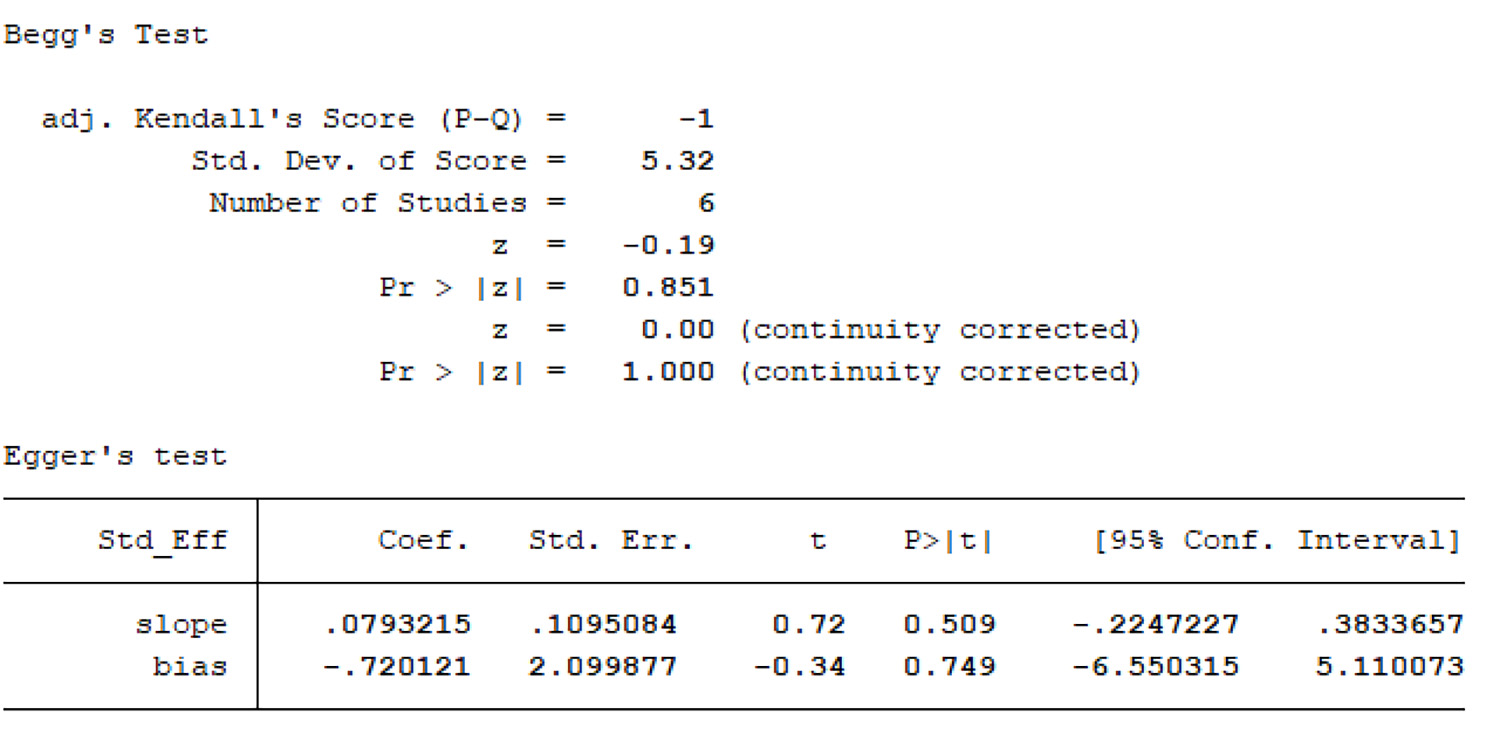

Supplement: Supplementary file 1 [file Data_Sheet_1.zip › Supplementary Material 2/Incidence of adverse reactions.jpg]

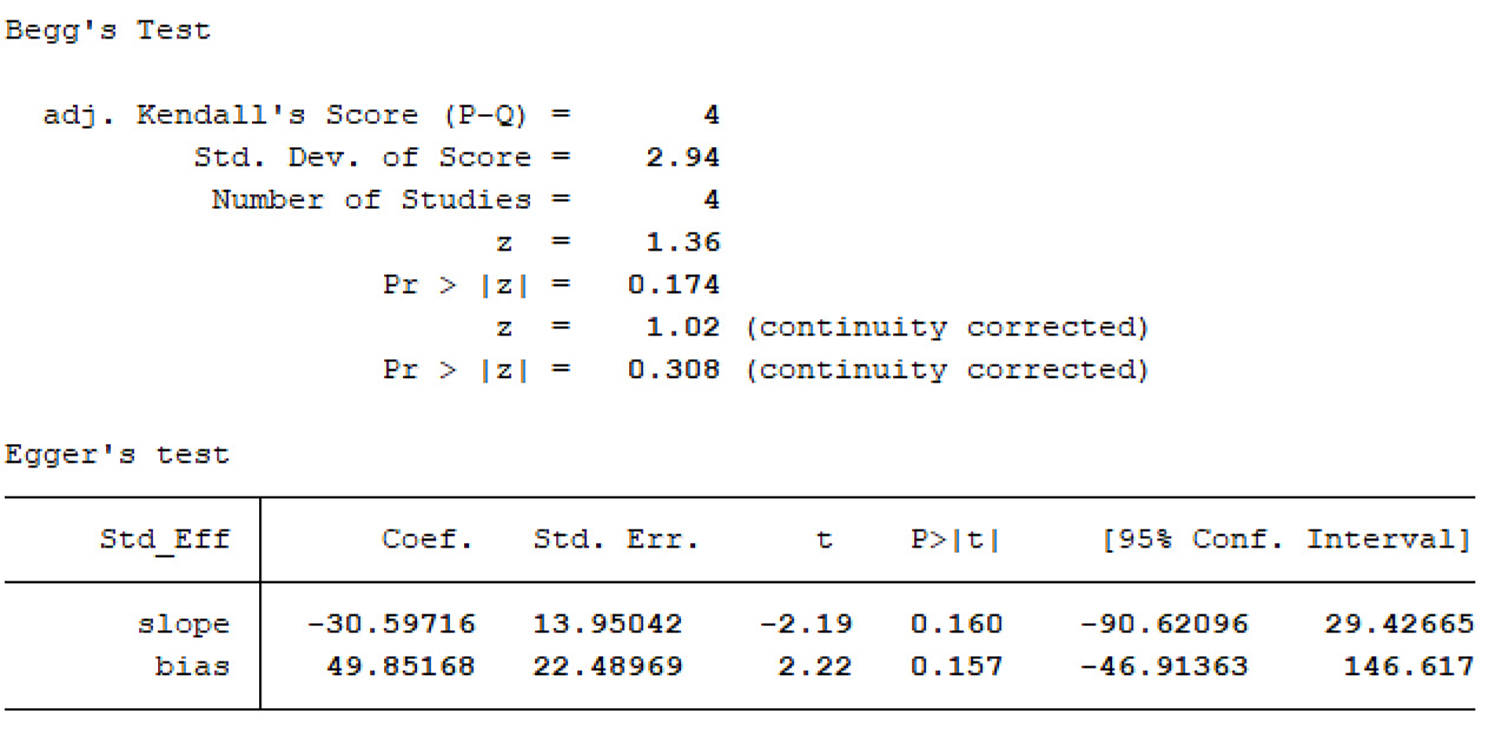

Supplement: Supplementary file 1 [file Data_Sheet_1.zip › Supplementary Material 2/Nerve conduction velocity.jpg]

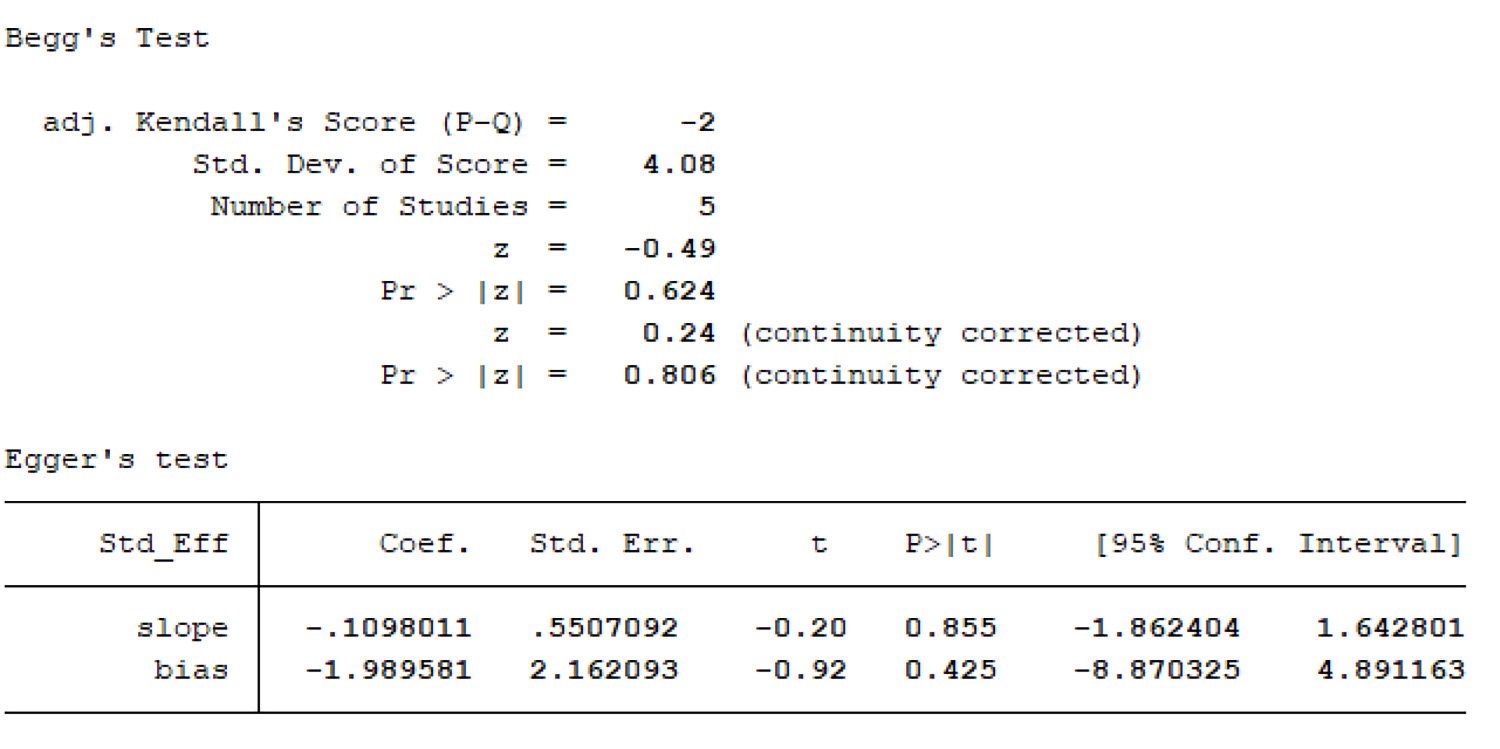

Supplement: Supplementary file 1 [file Data_Sheet_1.zip › Supplementary Material 2/Pain intensity.jpg]

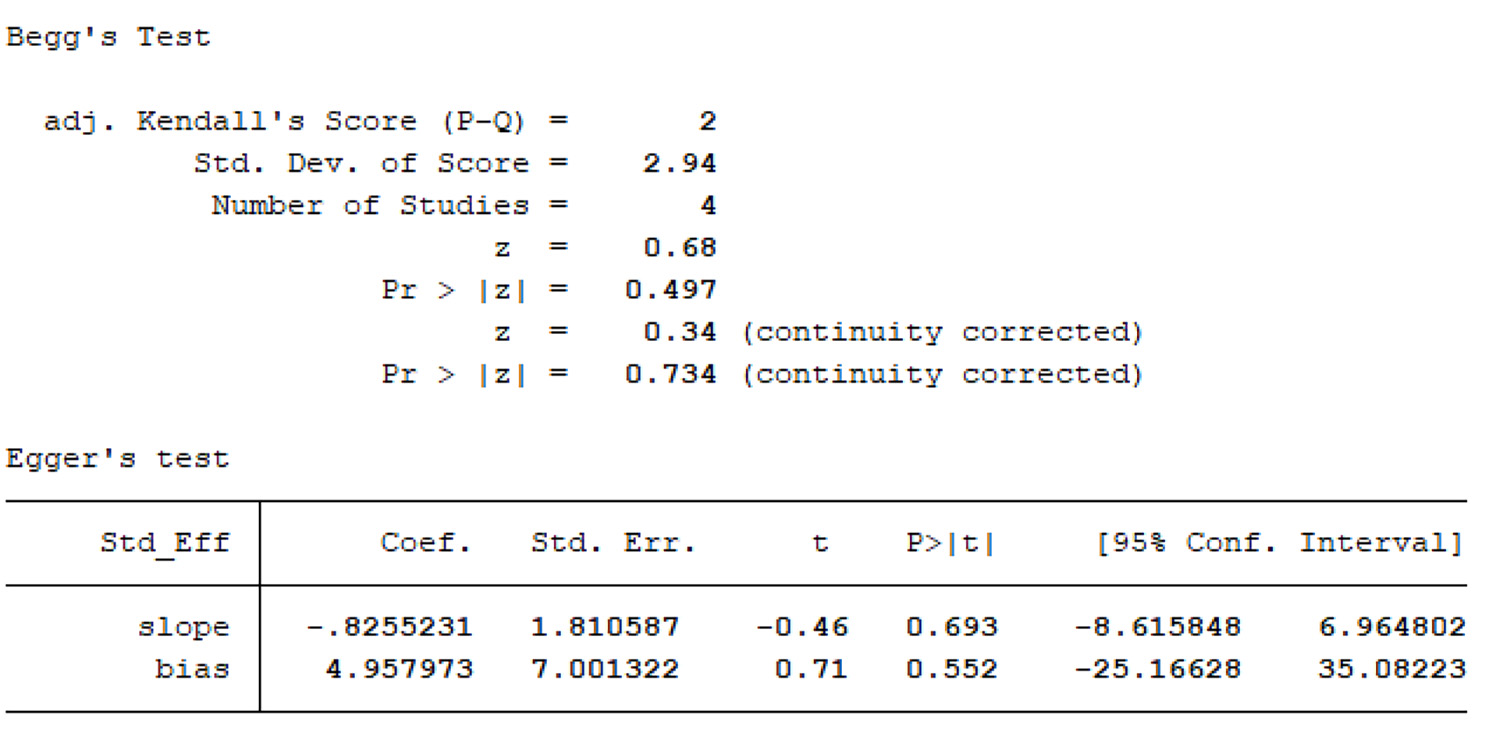

Supplement: Supplementary file 1 [file Data_Sheet_1.zip › Supplementary Material 2/Quality of life score.jpg]
